# Signal Amplification Strategy Based on TiO_2_-Nanotube Layers and Nanobeads Carrying Quantum Dots for Electrochemiluminescent Immunosensors

**DOI:** 10.1002/open.201300003

**Published:** 2013-04-12

**Authors:** Zhi-Da Gao, Qian-Lan Zhuang, Yan-Yan Song, Kiyoung Lee, Patrik Schmuki

**Affiliations:** [a] College of Sciences, Northeastern UniversityShenyang 110004 (P. R. China) E-mail: yysong@mail.neu.edu.cn; [b] Department of Materials Science and Engineering, WW4-LKO, University of Erlangen-NurembergMartensstrasse 7, 91058 Erlangen (Germany) E-mail: schmuki@ww.uni-erlangen.de

**Keywords:** electrochemiluminescence, quantum dots, signal amplification, TiO_2_ nanotubes

## Abstract

Self-organized TiO_2_-nanotube layers can be used for immunoassay-type sensing in combination with amplifying CdTe labels in a direct and very sensitive electrochemiluminescent (ECL) configuration. Key properties for this method are the conductivity of the TiO_2_ nanotubes, and their transparency for light emitted from the CdTe labels at approximately 2.4 eV. To demonstrate the potential of this platform, we constructed a sandwich-type immunoassay onto the TiO_2_-nanotube wall with a layer of (3-aminopropyl)triethoxysilane as the cross-linker for antibody immobilization. For the counter part of the sandwich, we created an amplification system consisting of TiO_2_ nanobeads carrying the secondary antibody and multiple CdTe quantum dots (multiQD). For antigen (IgG) detection, we find that this combination of 3D transparent electrode with multiQD labels allows for an ECL detection limit of 0.05 pg mL^−1^ and a linearity of the signal in the range of 0.1–10^8^ pg mL^−1^.

## Introduction

Immunoassays based on the specific antigen/antibody (Ag/Ab) recognition represent one of the most important quantitative techniques that are contemporarily applied in many fields of chemical analyses, including environmental monitoring, food safety, and clinical diagnosis.^1^ Due to the highly specific recognition, often sandwich-type immunoassays Ab_1_/Ag/Ab_2_* are used, where the detection labels (*) may be enzymes,^1^ fluorescence dyes,^2^ metal particles,^3^ quantum dots,^4^ etc. The detection limit of this approach is to a large extent determined by the amount of captured signal labels per recognition event, and, of course, the intrinsic sensitivity of the label detection concept. A common strategy to enhance the sensitivity of an immunosensor is to increase the amount of captured label per binding event, thus reaching signal amplification—for example, by using multiple fluorescence tags on a binding platform.^5^–^7^ For a direct detection, an increasingly used read-out strategy is based on electrochemical techniques, and here in particular electrochemiluminescence (ECL) approaches have attracted wide interest for their advantageous features, such as good portability, low cost, and low background noise.^8^ In this case, typically, visible light emission from Ru(bpy)^3+^, luminol, or quantum dot (QD) labels (e.g., CdX, X=Te, S, Se) in presence of co-reactants (e.g., H_2_O_2_, C_2_O_4_^2−^, S_2_O_8_^2−^ ions) is registered while applying a suitable electrochemical potential. For polarization, typically gold or glassy carbon electrodes that carry the immobilized immunoassay are used. To increase the sensitivity of electrochemical sensors, electrodes are built with a 3D nanoscale geometry to establish more binding sites for Ab. Typically mixtures of silica, carbon, gold and nafion nanoparticles are used, where gold and carbon provide the conductivity within the electrode. For ECL based electrodes, this carries the inherent drawback of a partially blocked light exit path (e.g., by carbon or gold) and thus partial sensitivity loss.

In the present work, we show that a promising alternative to conventional 3D nanostructured electrodes is provided by self-organized TiO_2_ nanotube (TiNT) structures. In contrast to many other oxides, TiO_2_ directly provides the required conductivity and transparency. Due to its band gap of *E*_g_≍3.2 eV, it is transparent for the visible emitted light in the ECL reaction of CdTe (2.4 eV) corresponding to approximately 510 nm. Moreover, TiO_2_ is regarded to reduce the injection barrier of electrons to CdX QDs due to its relative band-edge positions.^9^ The conductivity of TiNTs in the cathodic range (where ECL is generated) is due to its n-type behavior that under negative voltage leads to forward bias conditions. The aligned array structure provides a large surface area, minimizes electron and ion diffusion paths, and thus makes such structures ideal for their use in capturing, concentrating, and releasing load, or probing for molecules.

**Scheme 1:**
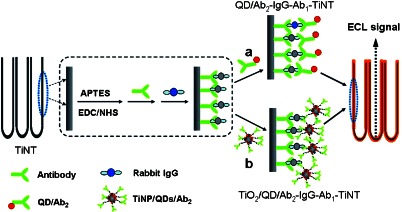
Construction of the immunosensors and ECL detection based on TiO_2_ nanotube (TiNT) arrays. Route a shows a sandwich-type immunoassay using a single CdTe label, and route b using a multiple label platform.

TiNT arrays formed by electrochemical anodization of titanium^10^, ^11^ can be tuned in geometry (i.e., diameter,^12^ aspect ratio^13^), “crystal” structure,^14^ electronic^15^ and biomedical properties^16^—even free standing membranes can be fabricated,^17^ and therefore very versatile nanoscale architectures can be built. Moreover, the distinct tubular feature provides tight nanocompartments, preventing lateral fluid spread—thus, the structures can be lithographically patterned, allowing sensor well arrays with minimum amount of analyte use and a high potential for further miniaturization of the immunosensor size. Due to the anodic formation from a titanium-metal substrate, another valuable feature is that the oxide tubes are directly back-contacted. Basically, TiO_2_-nanotubular-based electrochemical or optical sensors can be used directly, for example after functionalization with a suitable recognition element (as schematically shown in Scheme 1, route A). Under such conditions a maximum signal amplification, proportional to the area increase (compared to a nonporous electrode) can be expected.

To further increase the sensitivity, we combine the advantages of a TiNT electrode with a recently introduced principle of single recognition/multiple QD response,^5^ as outlined in Scheme 1. For this, we construct a sandwich-type immunoassay, where we first immobilize antibodies (Ab) on the tube wall, then capture the analyte molecules (IgG), and finally sandwich the specific analyte molecule with a TiO_2_ nanobead/multiQD/Ab unit. For maximum sensitivity, CdTe-QD-coated TiO_2_ nanoparticles (QDs/TiNP) were used as the signal amplification element. We show that this signal amplification element, in combination with the advantages of a TiO_2_-nanotube electrode, leads to an outstanding detection limit for the ECL technique.

## Results and Discussion

**Figure 1:**
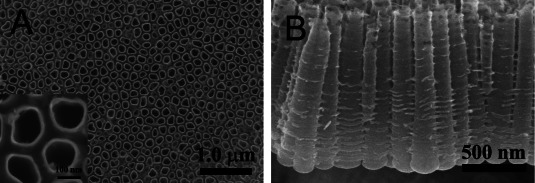
SEM images of the TiO_2_-nanotube layers used in this work: A) top view and B) cross-sectional view.

For preparation of the antibody-coated TiO_2_-nanotube (Ab_1_-TiNT) working electrodes, we used a self-organized TiO_2_-nanotube (TiNT) layer with individual tube diameters of 150±15 nm and lengths of 1.0±0.2 μm (Figure 1 A,B; details on the preparation are given in the Experimental Section). The tube walls were decorated by a saturated monolayer of (3-aminopropyl)triethoxysilane (APTES)^19^ and then reacted with the antibody (Ab_1_) in the presence of 1-ethyl-3-(3-dimethylaminopropyl)carbodiimide hydrochloride and *N*-hydroxysuccinimide (EDC/NHS). To characterize the coupling process, electrochemical impedance spectroscopy (EIS) was used. Spectra were recorded at different stages in a solution of 0.1 m KCl containing 5.0 mm Fe(CN)_6_^3−/4−^ (see Figure SI-1 in the Supporting Information). Under present conditions, the diameter of the semicircle represents the electron-transfer resistance (*R*_et_) of the Fe(CN)_6_^3−/4−^redox probe. The EIS of the neat TiNTs shows an almost straight line (curve a) that is characteristic of a diffusion-limited step in the electrochemical process. When the TiNT surface is modified with functional linker molecules, that is, APTES (curve b) or EDC/NHS (curve c), the electron-transfer resistances increase accordingly. When Ab_1_ molecules are bound onto the nanotube wall using acylamide binding, *R*_et_ further increases (curve d) due to the insulating protein layer. After the electrode is incubated with rabbit immunoglobulin G (RIgG), and after binding of the second antibody (Ab_2_), *R*_et_ increases further (curve e and f, respectively).

TiO_2_ nanoparticles coated with quantum dots and modified with antibodies (TiNP/QDs/Ab_2_) were used as probe in the immunosensor. In order to fabricate this immunosensing probe, TiNPs were first modified with APTES, and then coated with CdTe QDs using acylamide binding. The successful synthesis of TiNP/QD hybrids is apparent from the color change of TiNP (see Figure SI-2 in the Supporting Information). After coating with CdTe QDs, the TiNP suspension shows a light orange color, similar to the color of the CdTe in solution. Fluorescence spectra were also used to characterize the synthesis of the TiNP/QDs hybrids. The fluorescence emission from TiNP is negligible, whereas a strong fluorescence emission peak appears at 512.0 nm after CdTe QDs are attach onto TiNPs (cf. curve b and c in Figure SI-3 A in the Supporting Information). The peak position is consistent with the fluorescence emission peak of CdTe QDs (curve a), with a small red shift because of the increased diameter of CdTe when attached to the TiNPs. TEM images of TiNP/CdTe (see Figure SI-3 C) also demonstrate that the surface of TiNPs (see Figure SI-3 B) is surrounded by a layer of CdTe QDs. The particle size was also measured using a particle size analyzer (see Figure SI-4 in the Supporting Information). Clearly, the results are in agreement with the TEM images. Taken together, these results confirm that the CdTe QDs were successfully attached onto the surface of TiNP. Attachment of the TiNP can avoid the agglutination of CdTe, a common problem that occurs when small nanoparticles are used in biological systems.^20^ In the presence of EDC and NHS as activating reagents, carboxyl groups on CdTe QDs react with amino groups of the second antibody (Ab_2_) molecules, by which the antibodies are bound onto the TiNP/QDs (denoted as TiNP/QDs/Ab_2_). The attachment of Ab_2_ leads to a further decrease of the fluorescence intensity (see curve d in Figure SI-3 A).

**Figure 2:**
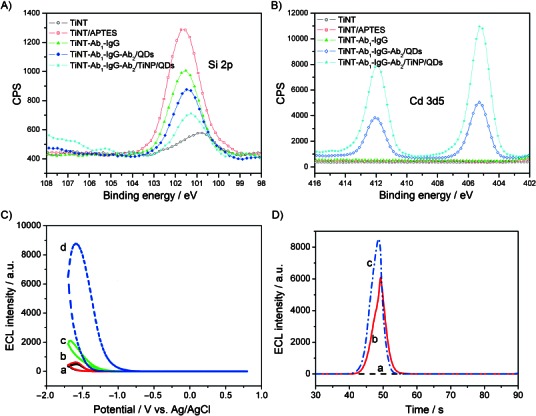
A) XPS Si 2p and B) Cd 3d5 peaks after the different assembly steps of the immunoassay on the TiNT surface (CPS=counts per second). C) ECL/potential curves of bare TiNT (a), IgG-Ab_1_-TiNT (b), QD/Ab_2_-IgG-Ab_1_-TiNT (c), and TiNP/QDs/Ab_2_-IgG-Ab_1_-TiNT (d) incubated in 1 μg mL^−1^ RIgG solution. D) ECL response of TiNP/QDs/Ab_2_-IgG-Ab_1_-TiNT in air-saturated 0.1 m PBS (pH 7.4) containing 0.1 m KCl without (a) and with (c) 0.1 m K_2_S_2_O_8_, and in 0.1 m PBS containing 0.1 m KCl and 0.1 m K_2_S_2_O_8_ after removing oxygen by nitrogen (b).

To test the two units introduced in this work, that is, the antibody-modified TiO_2_-nanotubes (Ab_1_-TiNT) and the quantum-dot-coated, antibody-modified TiO_2_ nanoparticles (TiNP/multiQD/Ab_2_), as effective immunosensors, we performed a sandwich-type immunoassay using TiNP/QDs/Ab_2_ as the immunosensing label, as outlined in Scheme 1 (route b). In a first step, Ab_1_-TiNTs were immersed in rabbit IgG (RIgG) solution for 2 hours to capture the antigen through the first immunoreaction. In a second binding immunoreaction, the immunosensing labels were captured within the nanotubes. We characterized the immunoarray assembly process to the TiO_2_ surface by XPS measurements of the TiNT surface after every attachment step (Figure 2 A,B). In the first step, a monolayer of APTES is attached to the surface—this is directly apparent in the Si 2p signal at 101.6 eV (Figure 2 A). Upon Ab_1_ attachment and RIgG coupling onto the titanium surface, a decrease of the Si 2p signal is observed. The attachment of Ab_2_-bound CdTe QDs is again very clearly visible in the XPS signals—most evidently from the large increase in the Cd 3d5 peaks at 412.1 eV and 405.3 eV (Figure 2 B). Obviously, the sample using TiNP/QDs/Ab_2_ immunosensing probes results in a higher increase of the Cd 3d5 signals and a larger decrease of the Si 2p signal compared to the sample using QDs/Ab_2_, which is in line with the concept that the hybrids of TiNP/QDs/Ab_2_ introduce more CdTe QDs into the nanotube arrays.

The sandwich-type immunoassay processes can also be followed by the electrochemiluminescence (ECL) characteristics of the electrode after the different modification steps. Figure 2 C shows the cyclic ECL curves for the immunosensor at each preparation stage. In 0.1 m phosphate-buffered saline (PBS), containing 0.1 m KCl and 0.1 m K_2_S_2_O_8_ as co-reactant, the ECL emission of both bare TiNT, Ab_1_-TiNT and RIgG-Ab_1_-TiNT, is negligible (curve a and b), which shows the ECL background to be very low. An obvious ECL signal is observed after the immunosensing labels are coupled to the nanotubes by the respective immunoreactions. Both attached immunosensing labels, QD/Ab_2_ (curve c) and TiNP/QDs/Ab_2_ (curve d), lead to considerable ECL.

To obtain a better understanding of the ECL generation, we carried out control experiments, and the results are plotted in Figure 2 D. No ECL emission peaks can be detected in PBS containing 0.1 m KCl without K_2_S_2_O_8_ (curve a), which confirms that S_2_O_8_^2−^ ions play a key role in ECL emission of QDs. The most plausible ECL mechanism is in line with previous reports.^18^ Briefly, when the potential is scanned in the negative direction, the CdTe QDs are reduced to form the anion radical (^.^CdTe^−^) by electron injection, and the S_2_O_8_^2−^ is reduced to produce a strong oxidant (^.^SO_4_^−^). ^.^SO_4_^−^ can react with ^.^CdTe^−^, by injecting a hole into the highest occupied molecular orbital (HOMO), opening a de-exitation path for the CdTe*QDs via luminescence emission. Furthermore, it should be noted that when dissolved oxygen is removed from the solution by N_2_ bubbling, a non-negligible ECL intensity decrease is observed (curve b, Figure 2 D) compared to air-saturated PBS containing KCl and K_2_S_2_O_8_ (curve c, Figure 2 D). These results demonstrate that oxygen also can be used to catalyze ECL emission of QDs. Thus, the following studies were carried out in an air-saturated PBS solution containing 0.1 m KCl and 0.1 m K_2_S_2_O_8_.

**Figure 5:**
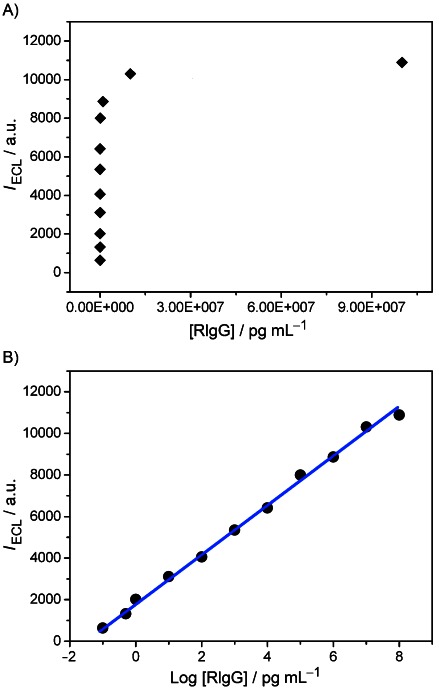
A) Influence of RIgG concentration on the ECL intensity from TiNP/QDs/Ab_2_-IgG-Ab_1_-TiNT immunosensor. B) Plot of ECL intensity versus logarithm value of RIgG concentration.

To explore the potential of the signal amplification strategies, we compared the multi-QD recognition label with a single-QD label, as illustrated in Scheme 1. In route A, QD/Ab_2_ labels are used as the recognition elements, whereas TiNP/QDs/Ab_2_ labels are used in route B. To quantify the response, we used a concentration of RIgG of 1 μg L^−1^. After the immunosensing labels are immobilized onto nanotube walls via the second binding events, at the same RIgG concentration the immunoassay using TiNP/QDs provides a 4-fold higher ECL emission magnitude (curve d, Figure 2 C) compared to the immunoassay using singleQD labels (curve c, Figure 2 C) on TiNT platform. If RIgG concentration is decreased to a lower value (i.e., 1 pg mL^−1^), the ECL signal could not be detected any more for the immunoassay based on singleQD labels. However, using the TiNP/QD labels, there is still easily detectable ECL (see also Figure 5). The results confirm that TiNP/QD/Ab_2_ labels can be introduced into the nanotubes successfully through the immunoreaction and the attached TiNP/QDs/Ab_2_ retains its ECL properties. These results also demonstrate the advantage to use TiNP/QDs/Ab_2_ labels as an effective recognition element for an ECL immunoassay based on TiO_2_ nanotube electrodes.

**Figure 3:**
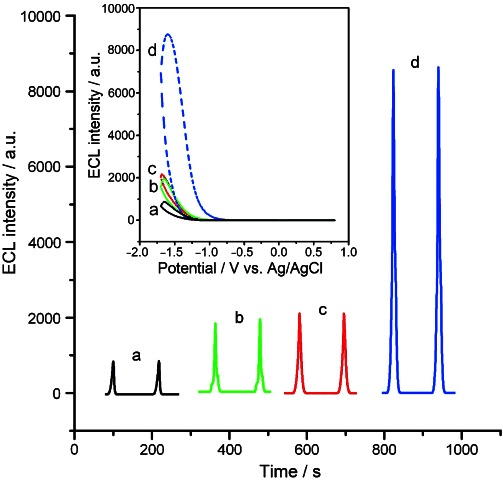
ECL response from QD/Ab_2_-IgG-Ab_1_-TiCL (a), TiNP/QDs/Ab_2_-IgG-Ab_1_-TiCL (b), QD/Ab_2_-IgG-Ab_1_-TiNT (c), and TiNP/QDs/Ab_2_-IgG-Ab_1_-TiNT (d) incubated in 1 μg mL^−1^ RIgG. Inset: the corresponding ECL/potential curves.

For comparison, we used a conventional flat TiO_2_ electrode (TiCL) and compared the ECL signal with the TiNT-based electrode at a RIgG concentration of 1 μg mL^−1^. As shown in Figure 3, for the nanotube-based TiNP/QDs/Ab_2_-IgG-Ab_1_-TiNT immunoassay (curve d), the ECL magnitude is 4.7-fold that of TiNP/QDs/Ab_2_-IgG-Ab_1_-TiCL immunoassay carried out on the flat electrode (curve b). This ECL emission intensity is similar to the result obtained from the TiNT-based immunoassay, however, using QD/Ab_2_ as recognition elements (curve c), thus indicating that both strategies (tubes and multiple QD label units) can contribute with a similar magnitude to the signal amplification. Clearly, the ECL emission from a TiO_2_ compact layer surface with QD/Ab_2_ as recognition elements (curve a) shows the lowest ECL intensity in Figure 3. This is in line with the findings that the ECL intensity of TiNP/QDs/Ab_2_-IgG-Ab_1_-TiNT is about 10.3-fold of the value obtained with QD/Ab_2_-IgG-Ab_1_-TiCL. The results demonstrate that the 3D structure of nanotubular arrays, due to its geometry, provides more opportunities for capturing antigen molecules per unit observation area. Moreover, the results show that there is sufficient space within the tubes for accommodating the bulkier recognition elements (i.e., TiNP/QDs/Ab_2_).

**Figure 4:**
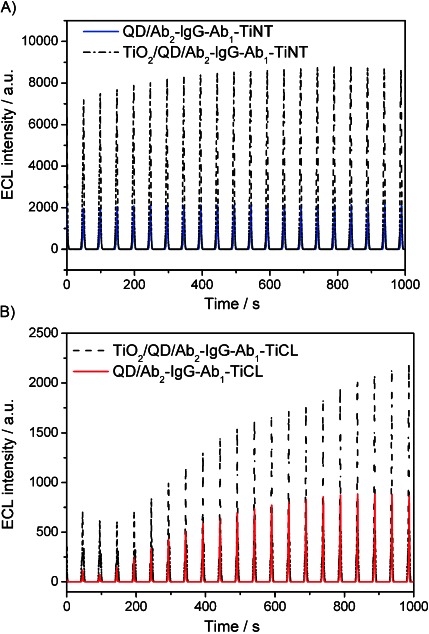
ECL emission from A) TiNT-based electrode and B) TiO_2_-compact-layer-based electrode by using QD/Ab_2_ and TiNP/QDs/Ab_2_ as immunosensing label upon 20 continuous cyclic scans in air-saturated 0.1 m PBS containing 0.1 m KCl and 0.1 m K_2_S_2_O_8_ at a scan rate of 100 mV s^−1^.

Moreover in Figure 4 A, the stability of the ECL emission was studied using the above-mentioned four kinds of immunoassays. Upon successive scanning between −1.7 and 0.8 V for 20 cycles, the ECL signals from the TiNT arrays (Figure 4 A) have a higher intensity and are more stable than the signals from the TiCT (Figure 4 B). These results suggest that the nanotubular array structure provides not only a more sensitive but also a more stable platform for the development of a high-sensitivity immunosensor.

To explore the detection limit and linearity of the sandwich-type immunoassay using TiNT as the platform and TiNP/QDs/Ab_2_ as recognition element, a RIgG concentration series was evaluated. As shown in Figure 5 A, the ECL emission intensities from TiNP/QDs/Ab_2_-IgG-Ab_1_-TiNT (after subtracting background signals) strongly increase with the RIgG concentration in the incubation solution. The ECL intensity increment was found to be logarithmically related to the concentration of RIgG in the range from 0.1 pg mL^−1^ to 1.0×10^8^ pg mL^−1^ (Figure 5 B). The linear equation is *I*_ECL_=1847+1171 log *c* with a detection limit of 0.05 pg mL^−1^ at a signal-to-noise ratio of three (R^2^=0.9986), where *I*_ECL_ is the ECL intensity and *c* is the RIgG concentration in pg mL^−1^. The obtained linear response range and the detection limit represent a considerable improvement from previous work,^18^ where best ECL-based immunosensors were constructed of carbon nanotubes and semiconductor QDs (yielding a linear response range from 20 pg mL^−1^ to 2.0×10^5^ pg mL^−1^ and detection limit of 1.0 pg mL^−1^).

## Conclusion

The combination of TiO_2_-nanotube arrays with TiNP/QDs/Ab_2_ as immunosensing label provides a highly sensitive immunosensing system for the detection of biomarkers at low level concentrations. Under optimized conditions, based on the dual-signal amplification mechanism, the nanotubular immunosensor presented here provides ultrasensitive electrochemiluminescence (ECL) detection of an antigen, with detection limits of 0.05 pg mL^−1^.

## Experimental Section

**Materials and Reagents**: Titanium foil (0.1 mm thickness, 99.6 % purity) was obtained from Advent Research. Rabbit immunoglobulin G (RlgG), goat antirabbit IgG antibody (Ab), and bovine serum albumin (BSA) were purchased from Boster Biological Technology (Wuhan, China). 1-Ethyl-3-(3-dimethylaminopropyl)carbodiimide hydrochloride (EDC), N-hydroxysuccinimide (NHS), (3-aminopropyl)triethoxysilane (APTES), and 3-mercaptopropionic acid (MPA) were purchased from Sigma–Aldrich. All other reagents were analytical grade and used as received. Phosphate-buffered saline (PBS) (0.1 mol L^−1^, pH 7.4) was used as supporting electrolyte. All aqueous solutions were prepared with deionized H_2_O.

**Apparatus**: The morphologies of the fabricated inorganic layers were characterized using a field-emission scanning electron microscope (Hitachi FE-SEM S4800, Japan). Transmission electron microscopy (TEM) was performed with a JEOL 2000 instrument operating at 200 kV accelerating voltage. The UV/Vis absorption spectra were measured on a UV-3900 spectrophotometer (Hitachi, Japan). The size of nanoparticles was analyzed by Zetasizer Nano ZS90 (Malvern, England). The ECL measurements were performed on a MPI-E multifunctional electrochemical and chemiluminescent analytical system (Remax, China). Electrochemical measurements were carried out using a CHI660D electrochemical workstation (CH Instrument, Shanghai, China). The TiNT samples (6 mm diameter), Pt wire and Ag/AgCl (3 m) electrode served as the working, counter and reference electrodes, respectively. Fluorescence spectra were recorded on Hitachi-F7000 spectrometer, X-ray photoelectron spectra (XPS) were recorded on a PerkinElmer Physical Electronics 5600 spectrometer using Al Kα radiation at 13 kV as excitation source. The take-off angle was 45°, with a resolution of 0.1 eV, using the binding energy of Ti 2p signal (458.0 eV) as the reference.

**Synthesis of TiO_2_** **nanotube arrays**: TiO_2_ nanotube (TiNT)) layers were formed by anodization of Ti. For this, Ti sheets of 0.1 mm thickness were degreased by sonication in acetone and EtOH, followed by rinsing with deionized H_2_O and drying in a N_2_ stream. The sheets were anodized in an electrolyte of glycerol/H_2_O (1:1 *v*/*v*) with 0.27 m NH_4_F at 30 V for 2 h, where the Ti foils were the working electrode and a Pt gauze served as the counter electrode. To grow the amorphous TiO_2_ compact layer (TiCT), the anodization was carried out at 20 V in 1 m H_2_SO_4_ for 20 min.

**Preparation of TiNP/QDs/Ab_2_** **and QD/Ab_2_** **immunosensing probes**: The preparation procedures of TiO_2_ nanoparticle (TiNP), quantum dots (QDs) and TiNP/QDs hybrids are described in the Supporting Information. To prepare antibody (Ab_2_)-modified immunosensing probes (TiNP/QDs/Ab_2_), a solution of the formed TiNP/QDs hybrids in PBS (1 mL, 0.1 m) was mixed with Ab in PBS (pH 7.4, 1 mL, 0.02 mg mL^−1^). Subsequently, freshly dissolved EDC in PBS (100 μL, 20 mg mL^−1^) and NHS in PBS (100 μL, 10 mg mL^−1^) were added. The mixture was gently stirred for 2 h at 4 °C. To block the unreacted and nonspecific sites, the obtained TiNP/QDs/Ab_2_conjugates were incubated with 2 wt % BSA for 4 h at 4 °C. The product was separated by centrifugation, re-dispersed in PBS (final volume: 2 mL), and stored at 4 °C when not in use. The preparation of QDs/Ab_2_ immunosensing probes is similar to the synthesis of TiNP/QDs/Ab_2_ described above. However, CdTe QDs (2 mL, 4 mg mL^−1^) was used instead of TiNP/QDs hybrids.

**Preparation of the antibody-modified TiNT arrays**: The as-prepared TiNT arrays were treated with APTES (10 mm) in toluene according to our previously reported grafting method to provide the amino-linking groups on the tube wall.^19^ The amino-functionalized TiNT arrays were rinsed with EtOH (3×) to remove unbound APTES molecules, and the tubes were incubated with EDC (20 mg mL^−1^) and NHS (10 mg mL^−1^) in PBS for another 30 min at RT. After washing thoroughly with deionized H_2_O, the TiNT arrays were incubated in Ab in 0.1 m PBS (pH 7.4, 0.5 mL, 20 μg mL^−1^) for 12 h at 4 °C, followed by blocking solution (2 % BSA, 100 μL) for 30 min at RT to block the remaining active sites against nonspecific adsorption. The resulting antibody-modified TiNT arrays (Ab_1_-TiNTs) were rinsed with PBS (pH 7.4) and stored at 4 °C when not in use.

**Preparation of immunosensor arrays**: The Ab_1_-TiNTs were incubated in a RIgG-containing PBS solution at RT for 2 h to capture RIgG (IgG-Ab_1_-TiNT). After washing thoroughly with PBS, the nanotube chips were immersed in a suspension of TiNP/QDs/Ab_2_ (0.5 mL) and incubated for 2 h at RT to introduce TiNP/QDs/Ab_2_ immunosensing probes into the nanotubes. The samples were rinsed thoroughly with PBS to remove nonspecifically bound probes.

**ECL detection**: The ECL signal was detected in PBS (0.1 m, pH 7.4) containing 0.1 KCl and 0.1 m K_2_S_2_O_8_ as a co-reactant. The ECL emission intensity corresponding to cyclic scan was recorded by a multifunctional electrochemiluminescence analyzer (MPI-E, Remax, China) at RT with the an ECL emission window placed in front of the photomultiplier tube biased at 550 V. The TiNT samples (top surface area: 28.3 mm^2^), Pt wire and Ag/AgCl (3 m) electrode served as the working, counter and reference electrodes, respectively. The cyclic ECL scan was carried out from 0.8 V to −1.7 V.

## Supplementary material

As a service to our authors and readers, this journal provides supporting information supplied by the authors. Such materials are peer reviewed and may be re-organized for online delivery, but are not copy-edited or typeset. Technical support issues arising from supporting information (other than missing files) should be addressed to the authors.
