# Supplementary material for: Signal Amplification Strategy Based on TiO2-Nanotube Layers and Nanobeads Carrying Quantum Dots for Electrochemiluminescent Immunosensors
Source: ChemistryOpen. 2013 Apr 12;2(3):93–8. doi: 10.1002/open.201300003 (PMC3703813; doi:10.1002/open.201300003)
Supplement: Supplementary file 1 [file open0002-0093-SD1.pdf]

ChemistryOPEN

Supporting Information

© 2013 The Authors. Published by Wiley-VCH Verlag GmbH & Co. KGaA, Weinheim

Signal Amplification Strategy Based on TiO_2-Nanotube Layers and Nanobeads Carrying Quantum Dots for Electrochemiluminescent Immunosensors

Zhi-Da Gao,[a] Qian-Lan Zhuang,[a] Yan-Yan Song,*[a] Kiyoung Lee,[b] and Patrik Schmuki*[b]

open_201300003_sm_miscellaneous_information.pdf

Supporting Experimental Procedure

Preparation of TiO_2 nanoparticles: TiO_2 nanoparticles (TiNP) were synthesized using a hydrolysis method with some modification.[1] Briefly, 1.1 mL of tetrabutyl titanate/ethanol (1:10) was dropped into 100 mL DI water under stirring at 20 ^{o}C. After continuously stirring for 3 h, the suspension was centrifuged, and mixed with 6.3 mL CH_3COOH (Ti^{4+}/CH_3COOH=1/2) under vigorous stirring for another 3 h. The resultant suspension was washed with ethanol for two times, and redispersed in ethanol. The diameter of the as-prepared TiNP was determined by particle size analyzer and TEM microscopy.

Preparation of CdTe quantum dots: The water-soluble CdTe quantum dots (QDs) were prepared using MPA as stabilizing agent according to a method that reported previously.[2] Briefly, 45.67 mg $CdCl_2 \cdot 2.5H_2O$ was dissolved in 80 mL of water, then 42 μL of MPA was injected into the solution and bubbled with pure N_2 for 30min. After that, a freshly prepared NaHTe solution (127.5 mg tellurium powder and 80 mg $NaBH_4$ in 1 mL of water at 0 ^{o}C) was injected into above solution. The solution was then heated at 80 ^{o}C for 6 h. The reaction mixture was purified by precipitation in absolute ethanol. Finally, the resulting CdTe was dispersed in 20 mL ethanol before use.

Preparation of QD coated TiNP: 0.4 mL of APTES was added into 2 mL TiNP (0.01 g mL^{-1}). After continuously stirring for 6 h, the mixture was centrifuged and washed with ethanol for four times. 25 mg of the amino-functionalized TiNPs were dispersed in a mixture of 1 mL of as-parepared CdTe QDs (4 mg mL^{-1}) and 1 mL EDC (20 mg mL^{-1}). After stirring for 12 h, the TiNP/QDs composites were centrifuged and washed for three times with water and redispersed in 1 mL of 10 mM pH 7.0 PBS solution.

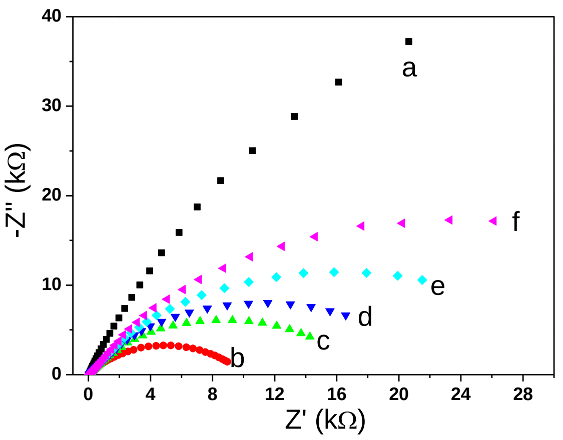

Fig. SI-1 Electrochemical impedance spectra for each immobilization step during the immunoassay process: (a) bare TiNT, (b) APTES decorated TiNT, (c) EDC/NHS/APTES decorated TiNT, (d) Ab_1-TiNT, (e) IgG-Ab_1-TiNT, and (f) TiNP/QDsAb_2-IgG-Ab_1-TiNT in 0.1 M KCl containing 5.0 mM Fe $(CN)_6^{3-/4-}$. The electrochemical impedance spectra were recorded at an open circuit potential with an signal amplitude of 5mV over a frequency range of 0.01 – 100 000 Hz. The RIgG concentration is 1 μg mL^{-1}.

Under present conditions, the diameter of the semicircle of electrochemical impedance spectroscopy (EIS) represents the electron-transfer resistance (R_{et}) of the $Fe(CN)_6^{3-/4-}$ redox probe. The EIS of the neat TiNTs shows almost a straight line (curve a) that is characteristics of a diffusion limited step in the electrochemical process. When the TiNT surface is modifed with functional linker molecules, i.e. APTES (curve b), EDC/NHS (curve c), the electron-transfer resistances increase accordingly. When the antibody molecules (Ab_1) are bound onto the nanotube wall via acylamide binding, R_{et} further increases (curve d) due to the insulating protein layer. After the electrode is incubated with RIgG and after binding of the second antibody, R_{et} increases further (curve e and f).

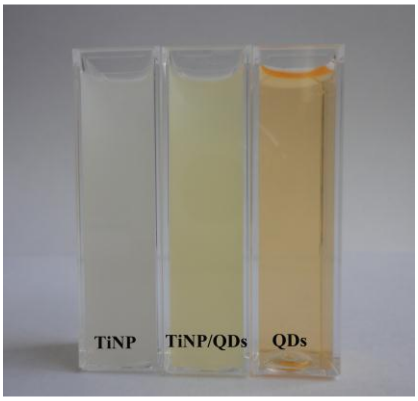

Fig. SI-2 Photos of suspension for TiO_2 nanoparticles (TiNP), CdTe quantum dots (QDs), and QDs modified TiO_2 nanoparticles (TiNP/QDs).

The success of TiNP/QDs hybrids is apparent from the color change of TiNP. After coating of CdTe QDs, the TiNP suspension shows a light orange color which is similar to the color of the CdTe in solution.

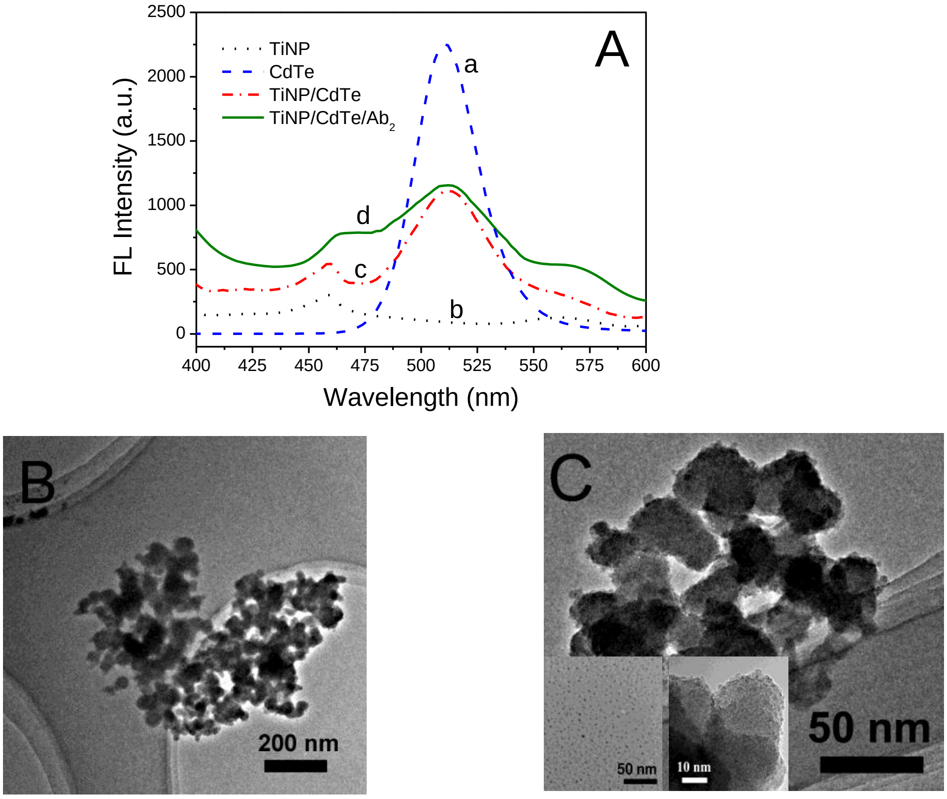

Fig. SI-3 (A): Fluorescence (FL) spectra of CdTe QDs (a), TiNP (b), TiNP/QDs (c), and TiNP/QDs/Ab_2 (d).: TEM images of (B) TiNP, and (C) QDs/TiNP, inset: CdTe QDs (left) and the magnification of QD/TiNP (right).

The FL spectrum (excited at 340 nm) of a 10-times dilution of the as-synthesized CdTe QDs solution shows a relatively narrow emission with the maximum intensity at 511.2 nm (curve a). The FL emission from TiNP is negligible (curve b), whereas a strong FL emission peak appears at 512.0 nm after CdTe QDs attach onto TiNPs (curve c). The peak position is consistent with the FL emission peak of CdTe QDs (curve a), with a little red shift because of the increased diameter of CdTe attached to the TiNPs. The attachment of Ab_2 leads to a further decrease of FL intensity (curve d).

In Figure SI-3B (inset, left), the TEM image shows the MPA-stabilized CdTe QDs of a very homogenous diameter of 2.0±0.3 nm. The TEM image of TiNP/CdTe demonstrates that the surface of TiO_2 nanoparticles is surrounded by a layer of CdTe QDs, the size of hybrids are 25±5 nm.

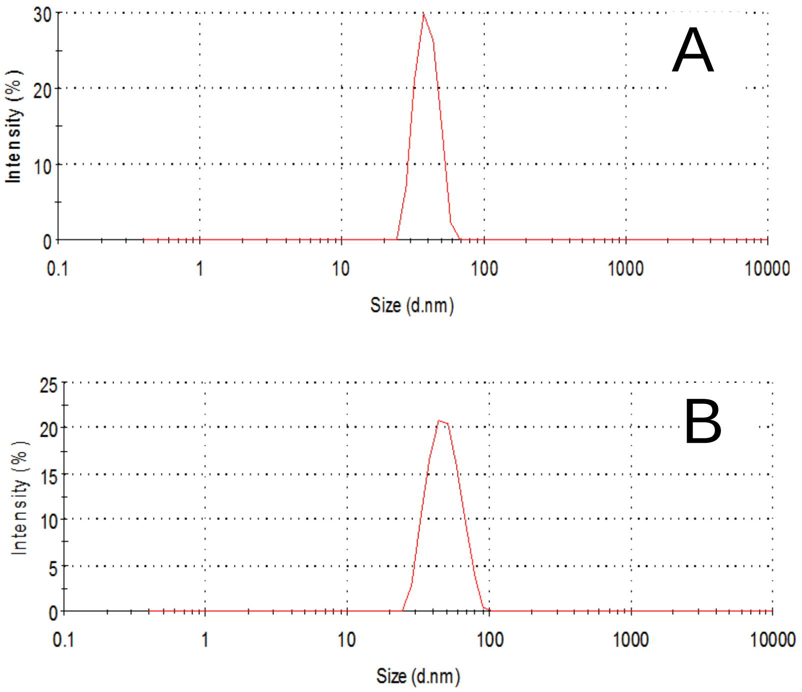

Fig. SI-4 Particle average size characterization of TiNP (A) and TiNP/QDs (B). The particle size of TiNP increases after the CdTe QDs attachment.

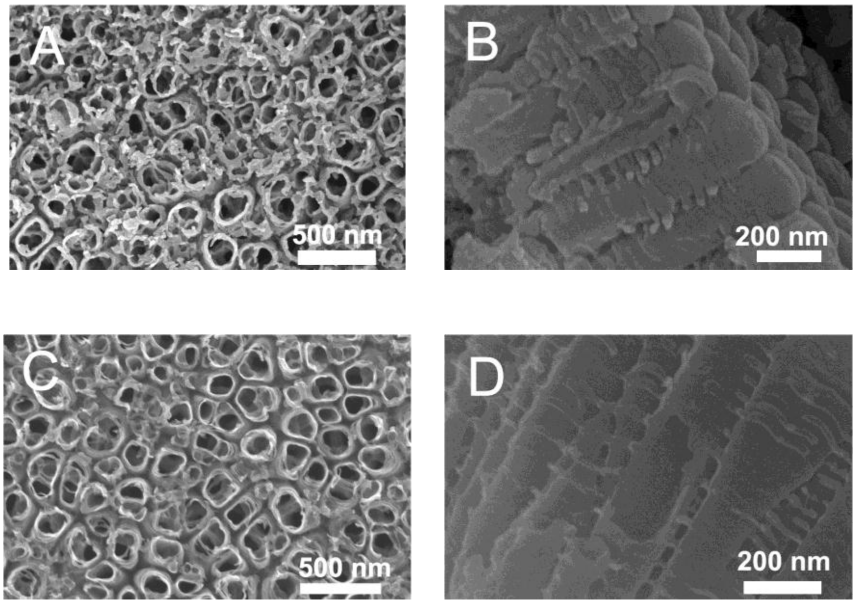

Fig. SI-5 SEM images of the TiO_2 nanotube layers after immunoassay:

TiNP/QDs/Ab_2-RIgG-Ab_1/TiNT (A) and (B), QDs/Ab_2-RIgG-Ab1/TiNT (C) and (D).

[1] J. H. Huang, L. Gao, J. Y. Chen, *J. Inorgan. Maters.* **1996**, *11*, 51-57

[2] Y. Y. Song, Q. L. Zhuang, C. Y. Li, H. F. Liu, J. Cao, Z. D. Gao, *Electrochem. Comm.* **2012**, *16*, 44-48.
